# Targeting USP42 induces DNA damage and inhibits cell growth in prostate cancer

**DOI:** 10.3389/fmolb.2025.1646331

**Published:** 2025-07-11

**Authors:** Yinghao Zhou, Chenchen Chen, Yibo Meng, Jianchao Ge, Shengkui Meng, Xillong Wang, Yaozong Xu, Guowei Shi, Wandong Yu, Xuetao Hu, Jun Zhang

**Affiliations:** Department of Urology, The Fifth People’s Hospital of Shanghai, Fudan University, Shanghai, China

**Keywords:** prostate cancer, deubiquitinase, DNA damage response, androgen recepter, olaparib

## Abstract

**Background:**

Prostate cancer (PCa) is one of the most common cancers in men worldwide. During its progression, deubiquitination-mediated alterations in biological processes play critical roles in tumor metabolism, stem cell characteristics, immune evasion, DNA damage repair, and chemoresistance. A comprehensive investigation of the deubiquitinases involved in PCa development holds significant clinical value as regards inhibiting tumor growth and overcoming drug resistance.

**Methods:**

Clinical databases were analyzed to identify differentially expressed deubiquitinases in PCa. Immunohistochemical analysis of PCa samples was used to evaluate USP42 expression in normal and tumor tissues. The effects of USP42 inhibition on PCa cell proliferation were assessed both *in vitro* and *in vivo* through MTT assays, colony-formation assays, and a subcutaneous xenograft tumor model in nude mice. The regulation of USP42 expression by the androgen receptor (AR) was investigated by culturing cells in low-androgen medium, modulating AR expression, and analyzing protein expression correlations through immunohistochemical staining of clinical samples and database analysis. The potential mechanisms underlying USP42-mediated effects on PCa cell proliferation were explored using RNA sequencing and data-independent acquisition proteomics. In addition, γ-H2A.X detection, MTT assays, and colony-formation assays were conducted to evaluate the impacts of USP42 inhibition on DNA damage repair and the therapeutic efficacy of olaparib in PCa cells.

**Results:**

Knockdown of USP42 significantly reduced PCa cell growth both *in vitro* and *in vivo*. USP42 expression was elevated in PCa tissues compared with normal tissues. Further investigation confirmed that AR positively regulated USP42 mRNA and protein expression in PCa cells. Mechanistically, USP42 inhibition induced significant defects in DNA damage repair. Moreover, USP42 knockdown markedly enhanced the tumor-suppressive effects of olaparib when used in combination.

## 1 Introduction

Prostate cancer (PCa) is one of the most common malignancies in men, and its incidence and mortality rates are rising annually (Bray et al., 2024). Androgen deprivation therapy (ADT) is the primary treatment for advanced PCa; however, after a period of ADT, most patients develop castration-resistant prostate cancer (CRPC), often leading to further tumor progression (Watson et al., 2015). Current treatments for CRPC include novel endocrine therapies, docetaxel chemotherapy, poly ADP ribose polymerase (PARP) inhibitors, and others (Rebello et al., 2021). Notably, PARP inhibitors target the DNA damage repair (DDR) pathway and have shown superior efficacy in patients with DDR-related mutations (Mateo et al., 2015; Pritchard et al., 2016). However, our understanding of the regulatory factors that influence DDR defects in PCa remains incomplete, highlighting the need for further research.

Deubiquitinating enzymes (DUBs) play a crucial role in cellular physiological regulation by modulating protein ubiquitination levels, thereby influencing various biological processes such as signal transduction and transcriptional regulation. In PCa, numerous DUBs have been implicated in tumor development and progression (Dewson et al., 2023). For example, USP7 promotes tumor growth by stabilizing the androgen receptor (AR) (Chen et al., 2015) and FOXA1^7^, and USP10 maintains p53 protein levels and regulates epigenetic changes induced by the AR (Takayama et al., 2018). Our team has reported that USP16 promotes PCa progression by stabilizing c-Myc (Ge et al., 2021).

Genomic instability is a critical hallmark of cancer, and defects in the cellular DDR promote tumorigenesis by disrupting genomic stability. DUBs have also been widely reported to participate in DDR. For example, USP1 contributes to this process by deubiquitinating PARP1 (Nes et al., 2024), while USP16 works in concert with HERC2 to regulate DDR (Zhang et al., 2014). POH1 is involved in the repair of DNA double-strand breaks (Butler et al., 2012). Some studies have highlighted specific DUBs involved in DDR within advanced PCa (Lin and Jin, 2024). USP3 can stabilize and deubiquitinate SMARCA5, influencing the DNA damage response and chemotherapy resistance in PCa (Li et al., 2024). USP14 may overcome DDR defects in autophagy-deficient cells by directly interacting with RNF168 (Sharma et al., 2018).

USP42, a DUB, has emerged as a multifaceted regulator in cell biology. It was first discovered in a gene fusion with RUNX1 in acute myeloid leukemia (Paulsson et al., 2006). Subsequently, it was progressively elucidated that USP42 plays regulatory roles in the stability of p53 and the ubiquitination levels of H2B (Hock et al., 2011; Hock et al., 2014). Moreover, USP42 can regulate the activation of WNT signaling by protecting ZNRF3/RNF43 from ubiquitin-dependent clearance (Giebel et al., 2021). In oncological research, USP42 is overexpressed in gastric cancer and regulates cell proliferation (Hou et al., 2016). It also forms liquid droplets in the nucleus through liquid–liquid phase separation (LLPS), promoting lung tumorigenesis (Liu et al., 2021). USP42 mutation may play a pivotal role in familial non-medullary thyroid carcinoma (Teixeira et al., 2024). However, the function of USP42 in PCa remains unclear.

In this study, we screened DUB members that affect the proliferation of PCa cells. As a result, USP42 was found to be critical for the growth of PCa cells both *in vitro* and *in vivo*. Deletion of USP42 led to DNA damage in PCa cells. Importantly, USP42 was elevated in PCa tissues from clinical samples and was regulated by AR. Furthermore, USP42 deficiency significantly enhanced the efficacy of olaparib in PCa cells. In conclusion, our study implies that USP42 could be a potential target for PCa treatment.

## 2 Materials and methods

### 2.1 Cell culture

The LNCaP, 22RV1, DU145, and PC3 cell lines were generously provided by the Stem Cell Bank, Chinese Academy of Sciences (Shanghai, China). These cell lines were cultured in RPMI 1640 medium (L210KJ; BasalMedia, Shanghai, China) supplemented with 10% fetal bovine serum (S660JJ; BasalMedia), 1% penicillin/streptomycin (15070063; Gibco, Grand Island, NY, United States), and 1% HEPES (15630080; Gibco). The prostate normal cell lines BPH-1 and RWPE-1 were purchased from the Stem Cell Bank, Chinese Academy of Sciences and cultured in keratinocyte complete medium (C120JV; BasalMedia). The method used to establish the LNCaP_AI cell line was described in our previous study (Zhou et al., 2024). All cells were maintained at 37°C in a humidified incubator with 5% CO_2_.

### 2.2 Plasmids and lentiviral infection

The short hairpin RNA (shRNA) sequences for each gene are detailed in Table 1. All shRNA sequences were cloned into the pLKO.1 vector. Lentiviruses targeting each gene were produced by transfecting HEK293T cells with the shRNA constructs, psPAX2 (#12260; Addgene, Watertown, MA, United States), and pCMV-VSVG (#8454; Addgene) using PEI 40K (G1802; Servicebio, Shanghai, China). For AR overexpression, full-length AR cDNA with a Flag tag was cloned into the pLVX-IRES-Puro vector between the XhoI and BamHI sites. Lentivirus-containing supernatants were collected at 48 and 72 h after transfection of 293T cells and used to infect 22RV1 and PC3 cells. Puromycin (5 μg/mL; Sigma-Aldrich, St. Louis, MO, United States) was used to select stable transformants.

**TABLE 1:** The short hairpin RNA sequence.

Target	Sequence (5ʹ to 3ʹ)
shCON	GCTCCGTGAACGGCCACGAGT
shUSP18	CACTGGCAGGAAACTGCATAT
shUSP22	CCTACCTGCTGTAAGATTATG
shUSP54	TTCATCCAGATGGTACATTAT
shMPND	CACCTACCTCGACAAGCTTAA
shPPPDE2	GCGGAAGATTCCTTCTTACAT
shUSP42-1	TGACCCTAAACGGTGCTAATA
shUSP42-2	CTTGATATTCGGCCATATATG
shAR-1	CGCGACTACTACAACTTTCCA
shAR-2	GATGTCTTCTGCCTGTTATAA

### 2.3 Cell growth and colony-formation assay

Cell growth evaluation was conducted using the MTT assay system. Cells (2,000 per well) were seeded into 96-well plates at 100 μL/well and incubated at 37°C under 5% CO_2_ until cell attachment (typically 24–48 h). Subsequently, 20 μL of MTT solution (final concentration of 0.5 mg/mL) was added to each well. The plate was gently mixed and incubated for 2–4 h to allow viable cells to reduce MTT to purple formazan crystals via mitochondrial dehydrogenases. After incubation, the medium was carefully aspirated without disturbing the cell layer or crystals, and 200 μL of solubilization solution (dimethyl sulfoxide) was added to dissolve the formazan crystals fully by shaking for 15 min. Finally, the optical density of each well was measured at 490 nm using a microplate reader. For the colony-formation assay, 22RV1 (1,000 cells/well) and PC3 cells (500–1,000 cells/well) were seeded in six-well plates with complete medium and cultured for 10–14 days, depending on colony size. Cells were then fixed in methanol for 10 min and stained with 0.1% crystal violet for 1 h.

### 2.4 Western blotting

Cells were gently washed three times with phosphate-buffered saline and lysed in lysis buffer. Protein samples were separated by sodium dodecyl sulfate–polyacrylamide gel electrophoresis and then transferred to a nitrocellulose membrane (10600001; Amersham, Marlborough, MA, United States). The membranes were blocked with 5% bovine serum albumin in Tris-buffered saline with Tween for three washes, then incubated with primary antibodies at 4°C overnight. Subsequently, the membranes were incubated with secondary antibodies corresponding to the primary antibodies at room temperature for 1 h and washed three times in Tris-buffered saline with Tween. Signal detection was performed using the Tanon Imaging System (Tanon-5200; Tanon, Shanghai, China). Antibodies used in the Western blotting assay were as follows: β-tubulin (SB-AB2002; ShareBio, Shanghai, China), AR (SC-56824; Santa Cruz Biotechnology, Dallas, TX, United States), USP42 (A15911; ABclonal, Wuhan, China), Vinculin (A2752; ABclonal), and γ-H2A.X (AP0099; ABclonal). All antibodies were diluted to the recommended concentrations according to the manufacturers’ instructions.

### 2.5 Real-time polymerase chain reaction (RT-PCR)

Total RNA was isolated using Total RNA Extractor (TRIzol) (B511311-0100; Sangon Biotech, Shanghai, China) in accordance with the manufacturer’s instructions, then converted to cDNA using the ABScript III RT Master Mix for qPCR with gDNA Remover (RK20429; ABclonal). Quantitative RT-PCR was performed using QuantStudio™ Real-Time PCR Software v1.7.1 (Applied Biosystems, Foster City, CA, United States) and the 2X Universal SYBR Green Fast qPCR Mix (RK21203; ABclonal). Relative gene expression levels were analyzed using the 2^−ΔΔCT^ method, with GAPDH serving as the internal control. The sequences of primers used to knock down the target genes are listed in Table 2.

**TABLE 2:** RT-PCR primer sequence.

Primer	Sequence (5ʹ to 3ʹ)
GAPDH-F	GTCAAGGCTGAGAACGGGAA
GAPDH-R	AAATGAGCCCCAGCCTTCTC
USP18-F	AGTCCCCGGCAGATCTTGAA
USP18-R	AAACCAACCAGGCCATGAGG
USP22-F	CTGCTCGCACCTGGGC
USP22-R	TACAGGACTTGGCCTTGCG
USP42-F	AGCCGGGTCAGAGTTGA
USP42-R	ATGAAGACACAGCACCCCAG
USP54-F	GTTGACTGTGCTGTCTGGCTA
USP54-R	TGAGCTTCGAGGTGCAAACA
MPND-F	CCAGTGACTATGGCATCCCC
MPND-R	ACTCCACCAGCAGCATCATC
PPPDE2-F	CTGAAGTTCTCTCCACGCCC
PPPDE2-R	GTCCCAGGCAGTCCTGTTAG

### 2.6 Immunohistochemistry (IHC)

Tissues were fixed in 10% buffered formalin for 24 h and embedded in paraffin. The paraffin-embedded tissues were sectioned and placed on charged glass slides, followed by hematoxylin–eosin or IHC staining using an IHC staining kit (HPA006752; Sigma-Aldrich), according to the manufacturer’s instructions. IHC scores were calculated using the formula: IHC score = intensity score × percentage score. The intensity score was based on staining intensity (0–4), and the percentage score was determined by the proportion of stained cells (0: 0%, 1: 1%–25%, 2: 26%–50%, 3: 51%–75%, and 4: 76%–100%). The antibodies used in the IHC assay were USP42 (HPA006752; Sigma-Aldrich) and AR (SC-56824; Santa Cruz Biotechnology). All antibodies were diluted to the recommended concentrations according to the manufacturers’ instructions.

### 2.7 Immunofluorescence

Cells were seeded at a density of 2 × 10^4^ cells/well onto glass slides in a 24-well plate and cultured for 24 h. The cells were then fixed with paraformaldehyde, permeabilized with 0.1% Triton X-100 for 10 min, incubated with γ-H2A.X antibody (AP0099, 1:200; ABclonal) at 4°C overnight, and subsequently incubated with FITC-labeled secondary antibodies for 1 h. Nuclei were counterstained with DAPI. Cell images were captured using a laser scanning confocal microscope (FV3000; Olympus, Tokyo, Japan).

### 2.8 Animal experiments

22RV1 cells (1 × 10^6^), infected with lentiviruses targeting USP42 or a control gene, were mixed with Matrigel (1:1, v/v) and subcutaneously injected into BALB/c nude mice (Si Pei Fu Biotech, Beijing, China). A tumor-free xenograft was defined as one that did not reach the flank. All mice were sacrificed after 30 days, and the xenografts were extracted, weighed, and photographed.

The experimental protocol was approved by the Experimental Animal Ethics Committee of the Department of Laboratory Animal Science, Fudan University. All animal experiment designs complied with the 3R principles (Replacement, Reduction, Refinement). The care and use of animals followed institutional guidelines. The BALB/c nude mice, which were specific pathogen-free, were purchased from Si Pei Fu Biotech. All mice were housed in a monitored environment at 23°C ± 1°C with 50%–60% relative humidity and a 12-h light/12-h dark cycle, with water and food provided *ad libitum*.

The mice were randomly divided into two groups, and different cells were subcutaneously injected into the flank. Tunnel handling was used to pick up the mice, and they were restrained using three fingers. Euthanasia was performed by CO_2_ asphyxiation followed by cervical dislocation. CO_2_ was dispensed from a commercial cylinder using a fixed-pressure regulator and inline restrictor, controlling gas flow within 30%–70% of the chamber volume per minute, in accordance with the 2020 American Veterinary Medical Association guidelines. CO_2_ flow was maintained for more than 60 s after respiratory arrest (which may take up to 5 min), followed by cervical dislocation to ensure euthanasia.

### 2.9 Bioinformatics analysis

Total RNA extracts from 22RV1 cells were subjected to RNA sequencing at Majorbio Biopharm Technology (Shanghai, China), and expression profiles were generated using the Majorbio Cloud Platform. Data-independent acquisition (DIA) proteomic analysis of 22RV1 cells was performed using an Orbitrap Astral mass spectrometer (Thermo Fisher Scientific, Waltham, MA, United States), and the resulting data were also analyzed on the Majorbio Cloud Platform.

Gene expression datasets of human PCa samples, including TCGA-PRAD (Hock et al., 2011; Hock et al., 2014) and GSE21034 (Hock et al., 2011; Hock et al., 2014), were obtained from The Cancer Genome Atlas (TCGA) database (http://portal.gdc.cancer.gov/) and the Gene Expression Omnibus database (http://www.ncbi.nlm.nih.gov/gds/), respectively. Gene set enrichment analysis (GSEA) was conducted using the GSEA software (version 4.2.2) provided by the Broad Institute (http://www.broadinstitute.org/gsea/index.jsp), with curated hallmark gene sets from the Molecular Signatures Database. The transcriptomic data have been deposited under BioProject ID: PRJNA1230828, and the mass spectrometry-based proteomic data are accessible via iProX ID: PXD061421.

### 2.10 Statistical analysis

All statistical analyses were performed using GraphPad Prism software Version 9.0 (GraphPad Software, San Diego, CA, United States). Quantitative data are presented as means ± standard deviation (SD). *P*-values of <0.05 were considered statistically significant.

## 3 Results

### 3.1 Identification of USP42 as a potential pro-oncogene in PCa development

**FIGURE 1:**
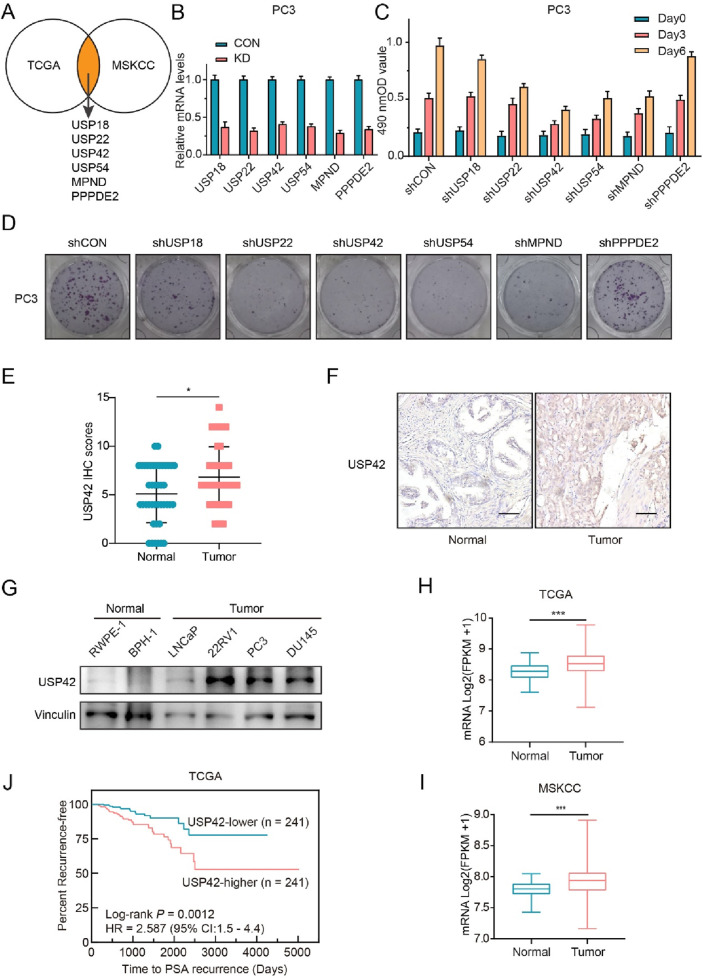
Screening and validation of key DUBs involved in PCa development. **(A)** Analysis of TCGA and MSKCC databases revealed significantly elevated expression of six DUBs in PCa tissues: USP18, USP22, USP42, USP54, MPND, and PPPDE2. **(B)** Validation of gene knockdown efficiency in PC3 cells by RT-PCR. Effects of DUB inhibition on PC3 cell proliferation assessed by **(C)** MTT assay and **(D)** colony-formation assay. **(E)** Statistical analysis and **(F)** representative images of USP42 IHC staining in normal (n = 42) and PCa (n = 43) tissues. **(G)** Representative Western blots showing USP42 expression in normal and tumor-derived prostate cell lines. **(H)** TCGA (normal = 52, tumor = 497) and **(I)** MSKCC (normal = 29, tumor = 150) database analyses showing significantly elevated USP42 expression in PCa tissues. **(J)** Kaplan-Meier analysis demonstrating a significant association between high USP42 expression and shorter PSA recurrence-free survival in patients with PCa. Data are presented as means ± SD. *P < 0.05, **P < 0.01, ***P < 0.001.

To explore potential targets that promote the emergence and progression of PCa, we initially screened the expression levels of all DUB members in tumor and normal tissues using two large public PCa clinical datasets (TCGA (Cancer Genome Atlas Research, 2015) and MSKCC (Taylor et al., 2010)). As shown in Figure 1A, six DUBs were identified for further investigation. We designed shRNAs targeting each gene, and RT-PCR was performed to confirm the knockdown efficiency of each shRNA in PCa cells (Figure 1B). The proliferation rates of PCa cells were then measured using the MTT assay (Figure 1C), and their colony-forming ability was also assessed (Figure 1D). Based on its effect on PCa cell growth, we selected USP42—which remains functionally uncharacterized in PCa development and progression—for further study.

We examined USP42 expression in normal (n = 42) and PCa tissues (n = 43) via IHC. The scores were significantly higher in PCa tissues than in normal tissues (Mann–Whitney test, *P* < 0.05) (Figure 1E). Staining intensity was weaker in normal prostate tissues than in PCa tissues (Figure 1F). Furthermore, we assessed USP42 expression levels in both normal and tumor-derived prostate cell lines. Compared with normal cells, USP42 expression was elevated in prostate tumor cells (Figure 1G). In addition, a Kaplan–Meier plot revealed a significant association between higher USP42 expression and shorter PSA recurrence-free survival in PCa patient cohorts (Figure 1J). Consistent with the IHC results, the mRNA level of USP42 was also upregulated in PCa tissues (Figures 1H,I). These findings imply that abnormally elevated USP42 expression may play an important role in PCa development and progression.

### 3.2 AR promotes expression of USP42 in PCa

**FIGURE 2:**
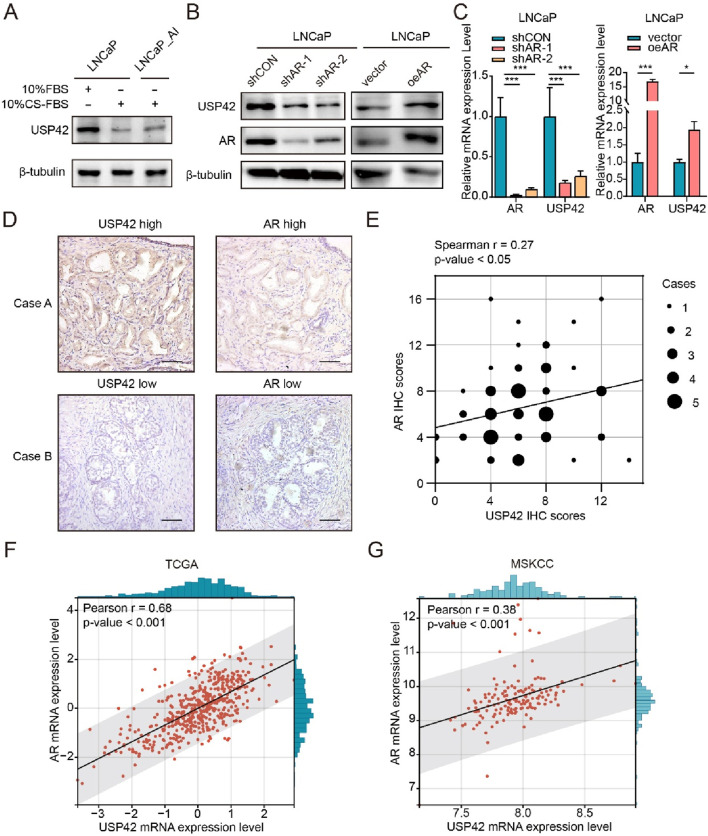
AR positively regulates USP42 expression. **(A)** Representative western blots showing changes in USP42 expression in LNCaP and LNCaP_AI cells cultured under low-androgen conditions. Effects of AR knockdown or overexpression on USP42 expression at the **(B)** protein and **(C)** mRNA levels. **(D)** Representative IHC staining images of AR and USP42 in serial sections of human PCa resection samples. **(E)** Correlation analysis between AR and USP42 expression in PCa tissues (n = 68). Correlation analysis of AR and USP42 expression using **(F)** TCGA (n = 498, the mRNA expression levels are presented as Z-scores of FPKM values) and **(G)** MSKCC (n = 152, the mRNA expression levels are displayed as Log2(FPKM+1)) datasets. Data are presented as means ± SD. *P < 0.05, **P < 0.01, ***P < 0.001.

ADT is the most common treatment for patients with PCa; therefore, we examined USP42 expression under low-androgen conditions. We found that in the androgen-sensitive PCa cell line LNCaP, USP42 was significantly downregulated after 3 days of low-androgen culture (Figure 2A). In the same experiment, we observed that USP42 expression in the androgen-independent cell line LNCaP_AI partially recovered (Figure 2A), implying that the presence of USP42 might contribute to PCa progression. Moreover, we knocked down AR using two different shRNAs or overexpressed AR via lentiviral transduction in PCa cells. The results show that USP42 expression decreased following AR knockdown and increased following AR overexpression (Figure 2B). This regulatory relationship between AR and USP42 was further confirmed at the transcriptional level (Figure 2C). To assess the correlation at the protein level, serial sections of human prostatectomy samples were analyzed using USP42 and AR IHC. USP42 and AR expression were significantly correlated in 68 human prostate tissue samples (Figures 2D,E). Additionally, to explore whether USP42 expression is linked to AR expression in PCa, we analyzed published human PCa datasets. Strikingly, a strong positive correlation was observed between USP42 and AR expression in two primary PCa cohorts (Figures 2E,F). Furthermore, USP42 expression levels showed a positive correlation with tumor stage Supplementary Figure S1A–C). These findings imply that USP42 may act downstream of AR in PCa.

### 3.3 USP42 knockdown decreased PCa cell growth *in vitro* and *in vivo*

**FIGURE 3:**
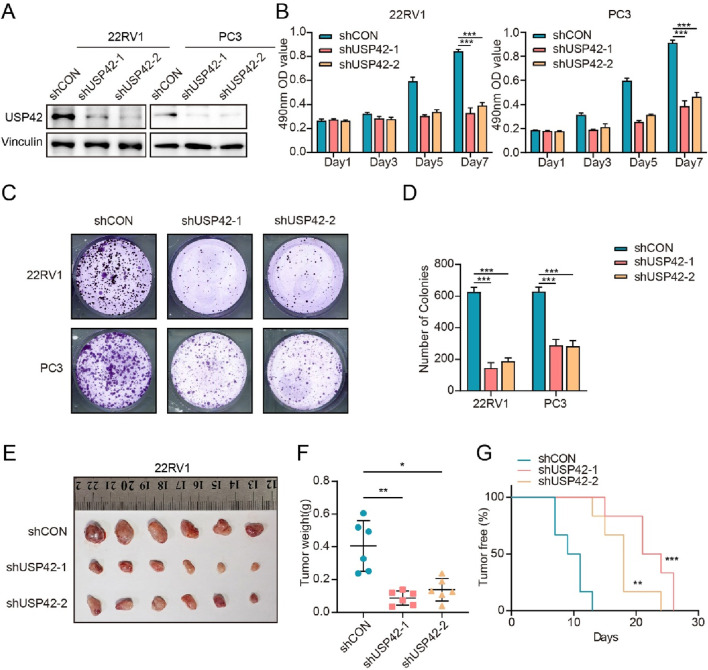
Knockdown of USP42 suppressed PCa cell proliferation *in vitro* and *in vivo*. **(A)** Construction and validation of stable USP42-knockdown 22RV1 and PC3 cell lines. Effects of USP42 knockdown on 22RV1 and PC3 cell proliferation assessed by **(B)** MTT assay and **(C)** colony-formation assay. **(D)** Quantification of colony formation. **(E)** Comparison of tumor volumes at 1 month after subcutaneous implantation of 22RV1 cells in nude mice (n = 6). **(F)** Statistical analysis of tumor weight (n = 6). **(G)** Statistical analysis of the tumor-free ratio in nude mice over time (n = 6). Data are presented as means ± SD. *P < 0.05, **P < 0.01, ***P < 0.001.

To investigate the role of USP42 in PCa cell proliferation, we silenced USP42 using two shRNAs targeting distinct sequences. The knockdown efficiency was confirmed by Western blotting (Figure 3A). Depletion of USP42 in 22RV1 and PC3 cells significantly inhibited cell growth (Figure 3B). Similarly, in the cell viability assays, USP42 knockdown markedly reduced the numbers of cell colonies (Figures 3C,D). Next, we subcutaneously injected PCa cells—with or without USP42 knockdown—into nude mice. During the 1-month follow-up, mice injected with USP42-knockdown cells exhibited markedly delayed tumorigenesis (Figure 3G). At the end of the study, all mice were euthanized and the xenografts were examined. Tumors were significantly larger (Figure 3E) and heavier (Figure 3F) in the control group than in the USP42-knockdown group, indicating that suppression of USP42 significantly inhibited PCa tumor growth *in vivo*. These results demonstrate that USP42 is required for proliferation of PCa cells both *in vitro* and *in vivo*.

### 3.4 USP42 deficiency causes DNA damage

**FIGURE 4:**
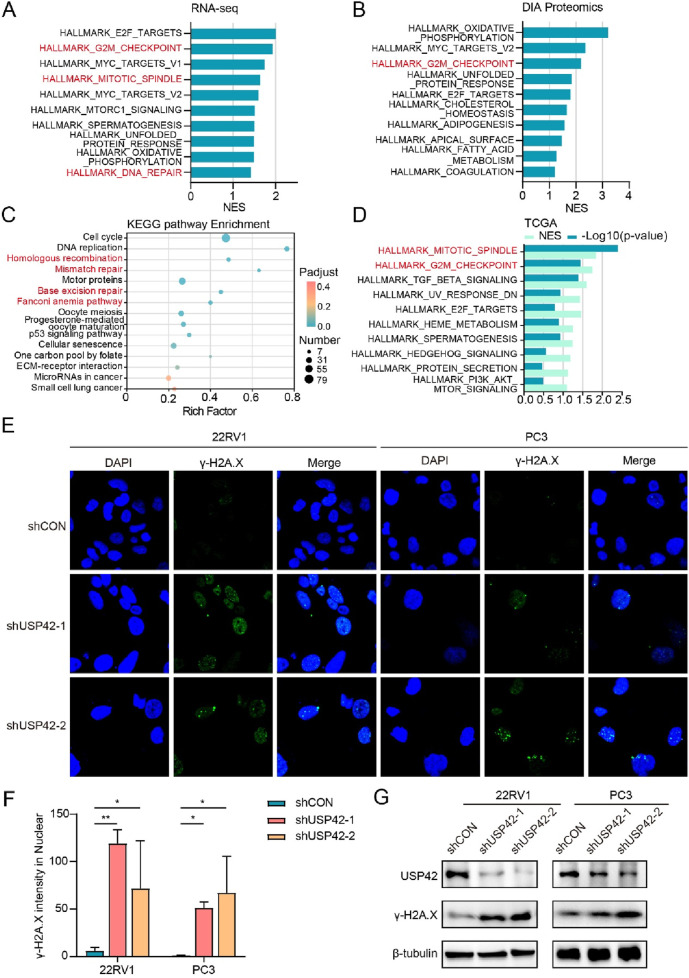
USP42 inhibition induced significant defects in DDR. GSEA plots from **(A)** RNA sequencing and **(B)** DIA proteomics analyses in 22RV1 cells following USP42 inhibition. **(C)** KEGG pathway analysis of downregulated genes in USP42-knockdown 22RV1 cells. **(D)** GSEA plot based on TCGA database analysis. γ-H2A.X levels in 22RV1 and PC3 cells upon USP42 inhibition assessed by **(E)** immunofluorescence and **(G)** Western blot. **(F)** Quantification of γ-H2A.X foci detected by immunofluorescence staining (n = 3). Data are presented as means ± SD. *P < 0.05, **P < 0.01, ***P < 0.001.

To investigate the possible mechanisms underlying the inhibitory effects of USP42 on PCa cell growth, we performed RNA sequencing and DIA-based proteomics following USP42 knockdown in 22RV1 cells. GSEA was used to analyze potential changes in biological states or processes. Gene sets related to the G2/M checkpoint and DNA repair were among the most highly enriched in control PCa cells (Figures 4A,B). Furthermore, KEGG analysis of downregulated genes in USP42-depleted PCa cells revealed significant enrichment in pathways related to DNA repair (Figure 4C). Similarly, relative normalized enrichment scores and *P*-values demonstrated that the G2/M checkpoint gene set was the most significantly enriched in patients with high USP42 expression (Figure 4D). Given these findings, we hypothesized that USP42 inhibition might be associated with increased DNA damage. To test this, we assessed γ-H2A.X, a marker of DNA damage, in PCa cells. USP42 knockdown resulted in a significant increase in γ-H2A.X foci (Figures 4E,F), and the protein levels of γ-H2A.X were also clearly elevated following USP42 depletion (Figure 4G). These data imply that USP42 deficiency induces DNA damage, contributing to its antitumor effects.

### 3.5 USP42 knockdown enhanced sensitivity of PCa cells to olaparib

**FIGURE 5:**
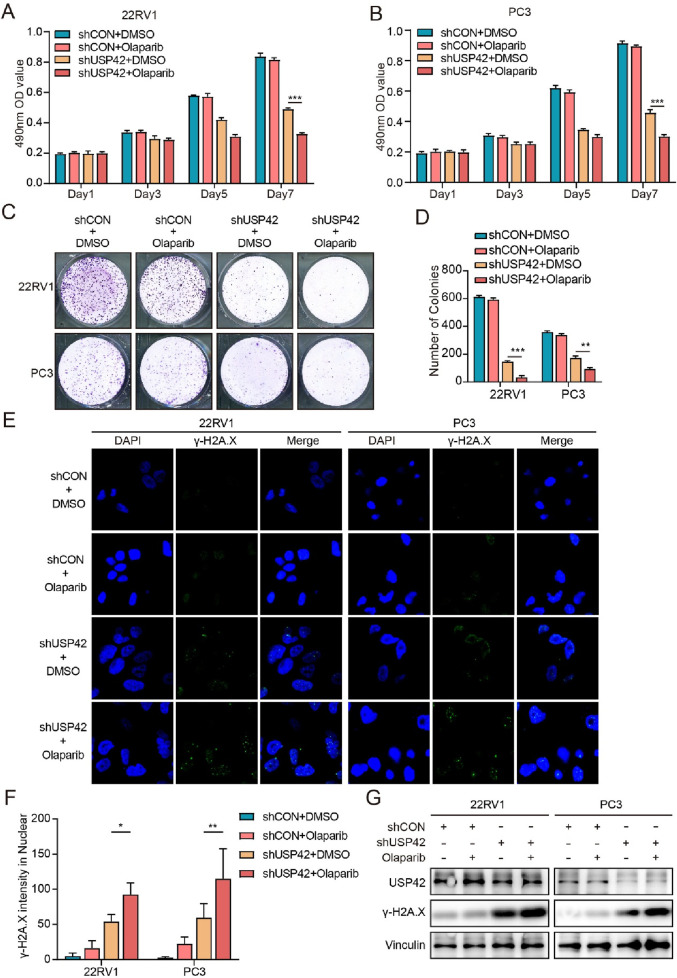
USP42 inhibition enhanced sensitivity of PCa cells to olaparib. Proliferation of **(A)** 22RV1 and **(B)** PC3 cells under different treatment conditions assessed by MTT assays. **(C)** Proliferation of 22RV1 and PC3 cells under different conditions assessed by colony-formation assays. **(D)** Quantification of colony formation. γ-H2A.X levels in 22RV1 and PC3 cells under different conditions assessed by **(E)** immunofluorescence and **(G)** Western blot. **(F)** Quantification of γ-H2A.X foci detected by immunofluorescence staining (n = 5). Data are presented as means ± SD. *P < 0.05, **P < 0.01, ***P < 0.001.

Olaparib, a PARP inhibitor, has been applied to treatment of metastatic PCa with DNA repair defects, and it has been confirmed that such patients can derive a survival benefit (Mateo et al., 2015). We investigated whether USP42 deficiency could enhance the efficacy of PARP inhibition in two olaparib-resistant PCa cell lines, 22RV1 and PC3. The combination of olaparib and USP42 knockdown led to more profound inhibition of cell growth than did either treatment alone (Figures 5A,B). Similarly, colony-formation assays showed that USP42 knockdown reduced cell survival under olaparib treatment (Figures 5C,D). Moreover, both the intensity of γ-H2A.X foci and its protein level were significantly increased in cells treated with both USP42 knockdown and olaparib (Figures 5D,F). These findings imply that the expression level of USP42 may influence the efficacy of olaparib in PCa.

## 4 Discussion

CRPC exhibits a variety of molecular alterations compared with hormone-sensitive PCa, potentially contributing to cancer progression through diverse mechanisms. Our study revealed that abnormal overexpression of USP42 may promote the development of PCa. However, the interpretation of USP42 expression patterns in this study should be tempered by the modest sample size derived from a single institution. Extending this work through prospective multi-institutional collaborations will be essential to establish population-level relevance. Additionally, reactivation and overexpression of AR have been extensively reported to drive castration resistance in PCa (Watson et al., 2015). Our findings suggest that AR regulates USP42 expression in PCa, implying that USP42 functions as a downstream effector in the pathogenesis of CRPC. ADT therapy promotes prostate cancer stem cell emergence, which is well known as a key driver of therapy resistance (Zhang et al., 2025). Intriguingly, our data reveal modulation of USP42 expression after ADT. Whether this molecular alteration contributes to cellular stemness warrants further mechanistic investigation. Subsequent experiments confirmed that depletion of USP42 significantly impaired the growth of CRPC cells both *in vivo* and *in vitro*, underscoring its critical role in sustaining growth signaling in CRPC.

Previous studies have indicated that AR is involved in activating the DDR response (Karanika et al., 2015). We observed that reducing USP42 levels led to DDR abnormalities, resulting in DNA damage, and inhibited tumor cell proliferation. Our findings imply that during AR-mediated castration resistance, USP42 may play an essential role in maintaining DDR integrity and contribute to the AR-driven enhancement of DDR. Furthermore, it has been reported that AR promotes PARP1 activation during PCa progression (Schiewer et al., 2012). Consistently, our experiments demonstrated that USP42 knockdown enhanced the inhibitory effects of PARP1 inhibitors on PCa cells.

Previous reports have shown that USP42 stabilizes p53 to facilitate recovery from mild or transient DNA damage (Hock et al., 2011). However, in our experiments using PC3 cells, which are p53-deficient, USP42 depletion still significantly affected tumor growth, indicating that its function in PCa is not entirely p53-dependent. Another study showed that USP42 regulates H2B ubiquitination, influencing gene expression in mammalian cells (Hock et al., 2014). However, when we examined ubH2B levels following USP42 knockdown in PCa cells, we did not observe significant changes (data not shown), implying that the mechanisms by which USP42 regulates DDR remain unclear. Through transcriptomic and proteomic analyses, we identified multiple DDR-related molecules whose protein expression was downregulated following USP42 depletion, without corresponding changes at the mRNA level. However, because of the large molecular weight of USP42, challenges in achieving effective overexpression and performing immunoprecipitation hindered further verification of its specific substrates in PCa. These questions warrant further investigation to uncover the precise substrates regulated by USP42 in DDR processes. Our study identifies USP42 as a potential determinant of olaparib sensitivity in castration-resistant prostate cancer (CRPC). Pharmacological co-targeting of USP42 may overcome olaparib resistance, thereby expanding the therapeutic applicability of PARP inhibition in prostate cancer patients.

## 5 Conclusion

We examined USP42 expression levels in clinical samples, cell lines, and public databases, demonstrating that USP42 is regulated by AR and contributes to CRPC progression. Further experiments revealed that targeting USP42 induced DNA damage in PCa cells and suppressed tumor growth both *in vivo* and *in vitro*. Additionally, we explored how USP42 depletion influences the efficacy of PARP1 inhibitors in PCa cells. Our findings imply that USP42 expression may correlate with olaparib sensitivity in PCa, highlighting USP42 as a potential therapeutic target for CRPC.

## Data Availability

The datasets presented in this study can be found in online repositories. The names of the repository/repositories and accession number(s) can be found in the article/Supplementary Material.

## Supplementary material

SUPPLEMENTARY FIGURE S1
USP42 expression levels positively correlated with tumor stage. **(A,B)** TCGA and **(C)** MSKCC database analyses showing significantly elevated USP42 expression in high stage PCa. Panel A and C: One-Way ANOVA, panel B: t-test Data are presented as means ± min to max. ***P* < 0.01, ****P* < 0.001.
