# Supplementary material for: Targeting USP42 induces DNA damage and inhibits cell growth in prostate cancer
Source: Front Mol Biosci. 2025 Jul 11;12:1646331. doi: 10.3389/fmolb.2025.1646331 (PMC12289470; doi:10.3389/fmolb.2025.1646331)
Supplement: Supplementary file 1 [file Image1.pdf]

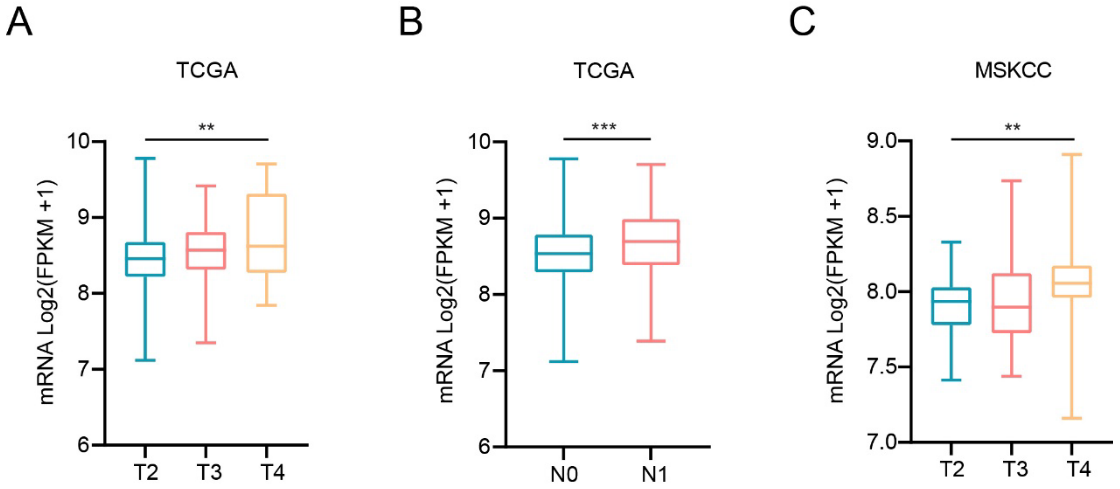

Supplemental Fig.1 USP42 expression levels positively correlated with tumor stage.

(A-B) TCGA and (C) MSKCC database analyses showing significantly elevated USP42 expression in high stage PCa. Panel A and C: One-Way ANOVA, panel B: t-test Data are presented as means ± min to max. ***P* < 0.01, ****P* < 0.001
